# IgG Avidity Test as a Tool for Discrimination between Recent and Distant *Toxoplasma gondii* Infection—Current Status of Studies

**DOI:** 10.3390/antib11030052

**Published:** 2022-08-15

**Authors:** Lucyna Holec-Gąsior, Karolina Sołowińska

**Affiliations:** Department of Molecular Biotechnology and Microbiology, Faculty of Chemistry, Gdańsk University of Technology, 11/12 Narutowicza Str., 80-233 Gdańsk, Poland

**Keywords:** IgG avidity, *Toxoplasma gondii*, diagnosis, toxoplasmosis, recombinant antigen

## Abstract

*Toxoplasma gondii*, an obligate intracellular protozoan parasite, is the causative agent of one of the most prevalent zoonoses worldwide. *T. gondii* infection is extremely important from a medical point of view, especially for pregnant women, newborns with congenital infections, and immunocompromised individuals. Thus, an accurate and proper diagnosis of this infection is essential. Among the available diagnostic tests, serology is commonly used. However, traditional serological techniques have certain limitations in evaluating the duration of *T. gondii* infection, which is problematic, especially for pregnant women. Avidity of *T. gondii*-specific IgG antibodies seems to be a significant tool for discrimination between recent and distant infections. This article describes the problem of diagnosis of *T. gondii* infection, with regard to IgG avidity tests. The IgG avidity test is a useful serological indicator of toxoplasmosis, which in many cases can confirm or exclude the active form of the disease. IgG antibodies produced in the recent primary *T. gondii* infection are of low avidity while IgG antibodies with high avidity are detected in the chronic phase of infection. Furthermore, this paper presents important topics of current research that concern the usage of parasite recombinant antigens that may improve the performance of IgG avidity tests.

## 1. Introduction

*Toxoplasma gondii* is an obligate intracellular protozoan parasite capable of infecting a wide range of endothermic vertebrates, including humans [1]. This parasite belongs to the phylum Apicomplexa, which contains numerous parasites of medical and veterinary significance including *Plasmodium*, *Cryptosporidium*, *Babesia*, *Neospora*, and *Eimeria* (causative agents of malaria, cryptosporidiosis, babesiosis, neosporosis, and coccidiosis, respectively) [2,3]. The apicomplexan parasites have complex life cycles with more than one infective form. Moreover, they are characterized by possession of a specific apical complex, composed of rhoptries, micronemes, polar rings, and a conoid, which participates in the invasion of the host cell [4]. The life cycle of *T. gondii* includes a sexual reproduction process that takes place only in the intestinal tract of definitive hosts (animals from the family *Felidae*, including domestic cats), and an asexual reproduction process that occurs in intermediate hosts (birds and mammals, including humans) [5]. During the life cycle of *T. gondii*, three developmental stages can infect cells: (i) tachyzoites (rapidly dividing); (ii) bradyzoites in tissue cysts (slowly dividing); and (iii) sporozoites contained in sporulated oocysts (environmental stage) [5,6]. All three stages are infectious for both intermediate and definitive hosts. Furthermore, the parasite may be transmitted from the definitive to the intermediate host, and vice versa, as well as between different definitive or intermediate hosts [5]. The sexual development of *T. gondii* leads to the formation of oocysts, which are excreted in the feces of infected cats. Favorable environmental conditions trigger sporulation, during which an oocyst divides into two sporocysts, each containing four sporozoites. Mature, sporulated oocysts are highly infective and any warm-blooded animal that ingests contaminated food or water becomes a host for the asexual cycle [7]. In the organism of the intermediate host, tachyzoites multiply rapidly and infect many types of host cells. After an acute infection, characterized by the dissemination of tachyzoites throughout the body, tissue cysts arise as a result of differentiation to the bradyzoite stage [5,6]. This stage of the parasite slowly grows within cysts located mainly in the central nervous system and muscle tissue of intermediate hosts [5,6]. *T. gondii* infection in humans is acquired through consumption of undercooked or raw meat containing tissue cysts (especially lamb and pork), ingestion of water or food contaminated by oocysts (excreted by felines) or by congenital transmission [8,9]. Moreover, rare causes of *T. gondii* infection include blood and blood product transfusion, or organ transplant [9].

Toxoplasmosis is not a new disease. The parasite was discovered in 1908, whereas *T. gondii* infection was first recognized in the late 1930s [9]. Early and acute infection is characterized by the presence of tachyzoites that are normally cleared by the host immune response. Next, when tachyzoites differentiate into bradyzoites, the infection becomes chronic. The bradyzoites can remain dormant within tissue cysts protected from the host immune response. In immunocompetent individuals, infection of *T. gondii* is usually asymptomatic. Mild clinical flu-like symptoms only occur in a small percentage of patients. However, this parasite poses a major threat to children infected in utero and immunodeficient individuals, especially to patients with acquired immunodeficiency syndrome (AIDS) and transplant recipients. Toxoplasmosis is believed to be one of the greatest causes of morbidity and mortality among the 10 most commonly occurring opportunistic infections in AIDS patients [10]. In immunocompromised patients, toxoplasmosis most often occurs as a result of reactivation of latent infection and presents neurological symptoms such as headache, disorientation, drowsiness, hemiparesis, reflex changes, and convulsions. As reactivated toxoplasmosis may be fatal, it is important to note that the incidence of a reactivation is relevant to the prevalence and concentration of IgG antibodies. In cases of high IgG avidity, reactivation is strongly suspected [11]. Toxoplasmosis diagnosis is also crucial for pregnant women as there is an established link between primary *T. gondii* infection during pregnancy and congenital infection. The highest risk of congenital transmission is found in mothers who are exposed to primary infection after conception, as opposed to those who have been infected before pregnancy [12]. What is more, the frequency of transmission and severity of congenital toxoplasmosis depend on the stage of pregnancy, in which maternal infection is acquired [13]. During the first trimester infection of *T. gondii* can lead to significant morbidity and mortality in the developing fetus, while if transmission of the parasite occurs during the third trimester, most cases are subclinical and result in asymptomatic infections or recurrent chorioretinitis [9]. It is therefore important to accurately distinguish acute and chronic stages of maternal infection in order to employ proper treatment. The most direct indicator of primary toxoplasmosis is documentation of seroconversion during pregnancy. However, this is rarely effective since women are not referred to preconception screening tests. Among various diagnostic methods available, serological tests are commonly employed for the diagnosis of *T. gondii* infection [14]. All serological methods used in the diagnosis of toxoplasmosis can be separated into two groups in order to optimize the interpretation of serological data. The first group includes fast or automated screening (e.g., hemagglutination and agglutination, enzyme-linked immunosorbent assay (ELISA), or chemiluminescence immunoassay (CLIA)), whereas the second group is used as confirmatory tests (e.g., dye test, immunofluorescence antibody test (IFAT), immunoblot, and immunosorbent agglutination assay (ISAGA)). Thus, a wide range of serological techniques are available for the detection of specific *T. gondii* immunoglobulins (Igs), which vary depending on the methodology, antigens used, and immunoglobulin isotype detected [15]. Interpretation of serological results is sometimes complex and may require complementary testing [16]. Furthermore, laboratory discrimination between acute and chronic stages of toxoplasmosis by commercially available assays is not easy. The diagnosis of recently acquired infection has been done traditionally by detecting specific IgM antibodies or by demonstrating a significant increase in specific IgG anti-*T. gondii* antibodies in paired serum samples collected at different durations of infection. However, a tendency of IgM against *T. gondii* to persist for a long time even at high levels has been verified in several studies [17,18,19]. What is more, natural IgM antibodies sometimes react with *T. gondii* antigens in the absence of the infection, leading to false-positive results [20]. Thus, the confirmation of specific IgM in a serum sample is an inadequate criterion for the diagnosis of recently acquired infection. Several reports have demonstrated the utility of detecting specific IgA or IgE antibodies for the recognition of the early phase of *T. gondii* infection [21,22,23,24]. These antibodies are produced during the first weeks of infection and disappear early [25]; however, the persistence of IgA and IgE antibodies following acute infection is still controversial. Currently, the distinction between recently and past acquired infection is based mainly on a measurement of IgG avidity of *T. gondii* specific antibodies. This method is cost effective as it not only optimizes toxoplasmosis screening but also minimizes expensive investigation methods and unnecessary treatments. It has been demonstrated that IgG avidity testing can confirm acute infection and differentiate between reactivations and primary infections using a single serum sample. This is especially important in pregnant and immunosuppressed patients. High IgG avidity results obtained in the first trimester of pregnancy exclude that the pregnant woman became infected with *T. gondii* during gestation [26]. Furthermore, it should be noted that low avidity is not a direct indication of a recent or past infection [27]. In some patients, low avidity may persist for as long as one year, which indicates that the avidity test is best used to rule out a recent infection in patients with high-avidity results [28,29]. In 2013, Villard et. al. [30] evaluated the performance of four commercially available Toxoplasma IgG avidity tests: Architect Toxo IgG Avidity (Abbott, Chicago, IL, USA), Vidas Toxo IgG Avidity (bioMérieux, Marcy-l’Étoile, France), Liaison Toxo IgG Avidity II (DiaSorin, Saluggia, Italy) and Platelia Toxo IgG Avidity (Bio-Rad, Hercules, CA, USA). The obtained results show that high avidity is distinctive for latent infection. However, low IgG avidity alone cannot be a definitive indicator of acute toxoplasmosis. What is more, some individuals receive borderline or equivocal avidity results. For these reasons, it is extremely important to search for, detect, and select new immunological markers for IgG avidity testing. Recombinant antigenic proteins of *T. gondii* might improve the performance of avidity tests to distinguish between acute and chronic phase of toxoplasmosis.

This article presents the current status of research concerning IgG avidity as an important diagnostic tool for discrimination between recent and distant *T. gondii* infections in humans and new diagnostic possibilities with the use of recombinant antigens.

## 2. Antibody Affinity and Avidity

For several decades it has been known that antibody avidity gradually increases after exposure to an immunogen [31,32]. The antibody response is generally characterized by a progressive increase in average affinity with time following immunization. Thus, the affinity maturation is the process whereby over time the binding affinity of IgG antibodies to the target antigens increases [31]. The mechanism of this process is driven by an interaction between B cells, T cells, antibodies, and antigens that leads to antigen driven B cell selection. As a result of this selection, the antibody-secreting B cells secrete IgG with increased affinity for binding specific antigens. The term ‘affinity’ is only applicable to uniform and immunologically monovalent antibodies. While the term ‘avidity’ (or functional affinity) refers to the accumulated strength of multiple affinities of individual non-covalent binding interactions, such as between antigenic epitopes and multiple antigen-binding sites of specific antibodies [33]. Thus, avidity differs from affinity, which describes the strength of a single interaction. IgG avidity is initially low after primary antigenic challenge and then by antigen-dependent clonal selection of B cells increases during the subsequent weeks and months. High avidity antibodies are found in circulation late after antigen stimulation. Therefore, the affinity maturation and associated changes in IgG avidity make possible a potential prediction of the period that has elapsed from the time of initial exposure to an agent of infectious disease. For these reasons, the avidity of IgG antibodies has been successfully applied in diagnostic studies of various infectious diseases [33]. Studies that were mainly conducted in the early to mid-1980s resulted in the appearance of the IgG avidity tests in clinical serology. Over the years, IgG avidity tests for *T. gondii* [34], rubella virus [35,36], cytomegalovirus [37,38], hepatitis viruses [39], varicella-zoster virus [40], and others were successfully developed and used in diagnosis, especially during pregnancy. Furthermore, during the past three decades, several different fully automatic or semi-automatic systems for IgG avidity determination have been developed.

## 3. IgG Avidity Assays in Serodiagnosis of *T. gondii* Infection

The IgG avidity test was developed by Hedman et al. in 1989 [34] to help discriminate between acute and chronic infection of *T. gondii* and since then has been further developed. This method measures the strength of an antibody binding to its target antigen, which requires close contact between complementary sites: epitope or antigenic determinant of antigen to the part of the antibody involved in binding, termed a paratope or combining site. The contact between these binding sites is based on weak and noncovalent interactions such as electrostatic interactions, hydrogen bonds, Van der Waals forces, and hydrophobic forces [41]. As mentioned before, this bond strength becomes progressively stronger over the duration of infection. Protein-denaturing reagents (e.g., urea) are used in a washing step with a buffer to dissociate the antibody–antigen complex and remove the low avidity antibodies. During this stage of the test, the presence of strongly binding antibodies can be detected. Currently, many companies in the world offer various kits for the determination of *T. gondii*-specific IgG avidity. There are also several automated systems that allow quick and easy definition of the ‘avidity degree’ (given as a percentage) or ‘avidity index’ AI (also known as ‘avidity coefficient’). AI calculations are performed in numerous ways. However, the easiest way to determine the avidity index is by calculating the ratio of signals obtained from two parallel measurements [39] according to the presented formula: AI = (signal with denaturation)/(signal without denaturation). Moreover, in IgG avidity tests, it is important to accurately define low, borderline or high IgG avidity. Commercial assays usually describe how the results are interpreted. In most cases, if the *T. gondii* infection occurred more than 3 months ago, the avidity is high (AI ≥ 60%), whereas in low avidity (AI ≤ 50%) infection is noted within the past 3 months [42]. Furthermore, borderline avidity (50% < AI < 60%) means that the acquirement of infection is not determined [42]. However, some problems and variance exist in the interpretation of obtained results. For example, several sources consider AI below 30% as low avidity, 30–40% as borderline avidity, and values above 40% as high avidity [43,44].

In less than 3 decades, measurement of IgG avidity has become a standard and basic method for dating of *T. gondii* infection. The determination of IgG avidity, after performing other tests (e.g., determination of IgG and IgM titers), in many cases allows confirmation or exclusion of active toxoplasmosis. Therefore, this test should be used in the diagnosis of the disease as a supplement to other diagnostic methods [42]. High avidity of specific IgG antibodies and the lack of early markers of active infection (IgM and/or IgA class antibodies) suggest chronic toxoplasmosis, and therefore allow discontinuation of testing subsequent serum samples, whereas low avidity of high IgG titers and the simultaneous presence of specific IgM and IgA antibodies make it possible to suspect active toxoplasmosis. On the other hand, cases of low titers of specific IgG with low avidity, questionable presence of IgM, and the presence or absence of IgA require serological testing of further serum samples and observation of antibody dynamics [25]. The reason for this is the fact that in some cases antibodies can mature for a long time, and therefore low avidity does not always indicate the early stage of the disease but only suspicion of a recently acquired infection, while high avidity of IgG-specific antibodies confirms infection acquired in the past. In addition, the determination of IgG avidity has some limitations. This test is useless in the case of reinfection, related to the reactivation of the parasite from the form of ‘dormant’ bradyzoites into ‘active’ tachyzoites, which may appear, among others, in AIDS patients [45]. In the organism of an infected individual, memory cells characterized by high avidity are already present. In addition, the usefulness of this test is also limited when testing congenital toxoplasmosis in newborns, as the avidity measured may be derived from maternal IgG antibodies that are transmitted through the placenta [46].

## 4. Recombinant Antigens in IgG Avidity Tests

**Figure 1:**
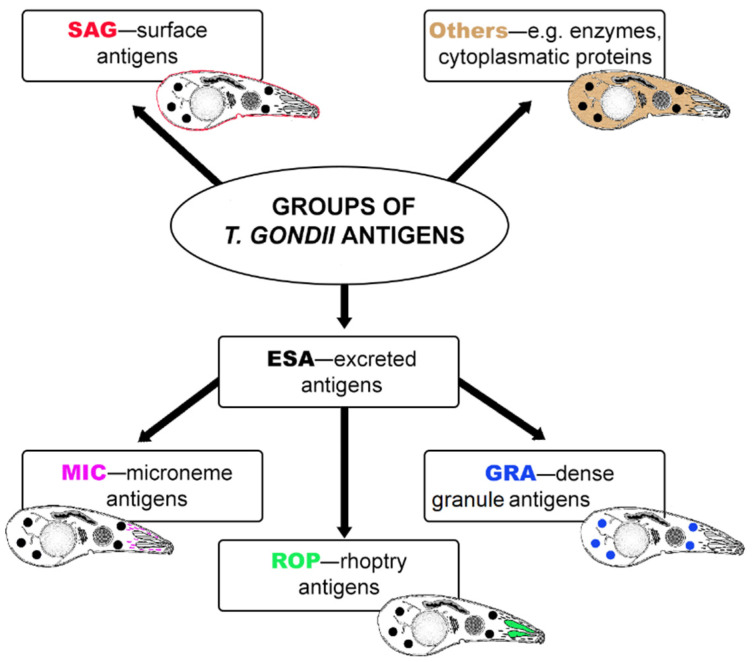
The main groups of *T. gondii* antigens.

At present, most serological tests for detection of *T. gondii* infection require the preparation of native parasite antigens from tachyzoites harvested from mice or tissue culture. The methods of obtaining native antigens may vary significantly between laboratories. Furthermore, this antigenic preparation obtained from tachyzoites may contain various nonparasitic materials from eukaryotic host cells or culture media [47]. Serological assays based on whole-tachyzoite antigens can cause false-positive results and are difficult to standardize. For the past few decades, recombinant *T*. *gondii* antigens have been considered an alternative source of proteins preparation that are less expensive and easier to standardize in serological tests. Many different recombinant antigens have been obtained from bacterial and eukaryotic expression systems and evaluated for their usefulness in serodiagnosis of *T. gondii* infection in humans [47,48] or domestic and farm animals [49]. The sensitivity and specificity of assays for the detection of specific antibodies depend on the antigen used. Thus, the rational selection of recombinant protein is crucial for obtaining antigenic preparation with the best diagnostic utility. Very often, for the diagnosis of toxoplasmosis, surface proteins or antigens of secretory organelles of the parasite that are important in its life cycle and during host cell invasion are selected. *T. gondii* proteins are localized in outer membrane, cytosol, and secretory organelles (Figure 1): the micronemes, the rhoptries, and the dense granules from where they are released to the forming parasitophorous vacuole (PV) during host cell invasion or to the tissue cyst [50,51,52]. The micronemes and the rhoptries discharge their contents from the apical end of the parasite, and the dense granules discharge from the apical, lateral, and posterior surfaces. The contents of the three secretory organelles have been known to be released sequentially according to a cascade mode [51,53,54]. The microneme proteins (MIC) are released first, upon contact with the host cells. These proteins are involved in host cell recognition and attachment [48]. The rhoptry proteins (ROP) are released next, and they may facilitate formation of the PV and mediate its clustering with host cell organelles [55]. The dense granule proteins (GRA) are exocytosed both during and after invasion into the PV. Following secretion, most of the GRA antigens appear as both a soluble and a membrane-associated form in the vacuole and they are thought to modify the environment within the PV, thereby functioning for intracellular survival and replication [56,57]. Moreover, these proteins constitute an important fraction of antigens that circulate in the bloodstream during the first hours following infection [58]. The surface of *T. gondii* is covered with a family of lycosylphosphatidylinositol (GPI)-anchored antigens (SAGs) and SAG-related sequence (SRS) proteins, most of which are members of the SAG1 or SAG2 families [59]. These proteins appear to play a role in host cell invasion, immune modulation, and/or virulence attenuation, although they may also provide protection needed by the parasite to survive in the environment [59].

In addition to fully automatic or semi-automatic systems, ELISA is the most frequently used method in clinical laboratories applied for diagnosis of *T. gondii* infection. This assay is still considered one of the most common techniques with high sensitivity and specificity in the quantitative detection of antibodies or antigens. For this reason, assays for measurement of IgG avidity are usually based on this technique. Moreover, Western blots or nitrocellulose strips are also commonly used. Initially, whole-tachyzoite antigens or excreted-secreted antigens were the primary testing antigenic preparations in these methods. However, due to the various limitations of avidity tests based on these antigenic preparation, new diagnostic tools that might improve the performance of avidity tests to distinguish between recent and past phases of toxoplasmosis are tested. In the past 20 years, several studies have reported the use of single recombinant antigens [44,60,61,62,63,64,65,66], mixture of proteins [44,67,68] or chimeric proteins [69] in the determination of IgG avidity (Table 1). All these antigens have been obtained in *Escherichia coli* expression systems and purified by affinity chromatography. Most of the papers concern the use of single antigenic preparations especially from dense granule proteins family, surface antigens group or rhoptry proteins family.

More than 20 years ago, Marcolino et al. [71] described the efficacy of a single antigen-based IgG immunoblotting avidity test. Two molecular markers (p38 and p60 antigens) for detection of low-avidity IgG in acute *T. gondii* infection were identified among a large group of tested proteins (p10, p16, p19, p23, p30, p32 (GRA6), p38, p40, p43, p54 (ROP2), p57, p60 (ROP1), p66, p70, p75, p83, and p97) prepared from the parasite suspension obtained from peritoneal exudates of previously infected Swiss mice. These studies showed that it is possible to identify single antigenic proteins that will be potential molecular markers. The next years of research focused on the use of various forms of recombinant proteins in IgG avidity tests. In 2003, Beghetto et al. [60] compared IgG avidity tests using a combination of four recombinant proteins (two dense granule antigens -GRA3, GRA7, microneme protein MIC3, and surface antigen SAG1) and whole-cell *T. gondii* lysate antigen (Vidas Toxo IgG Avidity, bioMérieux). The avidity index for IgG antibodies against a homogeneous mixture of recombinant antigens was correlated closely with the IgG avidity of antibodies against the lysed native antigens. Furthermore, the IgG avidity test based on MIC3 recombinant antigen highlighted the presence of low avidity IgG antibodies exclusively in sera collected within 2 months after primary infection. Therefore, this study showed that affinity maturation of antibodies against single epitopes represented by *T. gondii* recombinant antigens may follow a different pattern than for the native antigen lysate. In 2005, Pfepper et al. [61] used five recombinant antigens (ROP1, MAG1, SAG1, GRA7, and GRA8) onto nitrocellulose strips in a line assay (*recom*Line Toxoplasma). This test allows differentiation of reactivities and IgG avidities against individual recombinant antigens. Moreover, ROP1 recombinant protein was applied in avidity assays as a single antigen [63], as a component of mixtures of two recombinant proteins (ROP1 + SAG2, and ROP1 + GRA6) [67] and in the form of new chimeric proteins [69]. This antigen is a very important protein for the parasite as it is involved at the early stage of *T. gondii* invasion into host cells. ROP1 is secreted into the interior of the forming parasitophorous vacuole during parasite entry into host cells, and its expression is inhibited a few hours after the invasion [72,73]. The results obtained by Holec-Gąsior et al. [63] suggested the application of recombinant ROP1 in an IgG avidity assay can be useful for detection of acute stage of infection. When the ROP1 antigen was combined with SAG2 surface antigen or GRA6 dense granule proteins, IgG avidity ELISA using these mixtures allowed for a more accurate diagnosis, especially for individuals who appeared to be difficult to diagnose by means of commercial avidity assays [67]. Furthermore, in 2019 Ferra et al. [69] for the first time showed IgG avidity ELISA using next-generation recombinant chimeric proteins in the form of trivalent antigen (SAG2-GRA1-ROP1) and tetravalent antigens (SAG2-GRA1-ROP1-AMA1N, AMA1N-SAG2-GRA1ROP1, AMA1C-SAG2-GRA1-ROP1, and AMA1-SAG2-GRA1-ROP1). One of the above-mentioned chimeric recombinant proteins (AMA1-SAG2-GRA1-ROP1) showed the greatest potential for determining the avidity of IgG antibodies. Another antigen that has been frequently tested in the IgG avidity assay is the SAG1/P30 recombinant protein, which is the most immunodominant *T. gondii* antigen. As the most abundant surface antigen of *T. gondii* tachyzoites, SAG1 plays a crucial role in the process of attachment to host cells. It is also known for its lack of cross-reactivity to other antigens [70]. In 2007, Pietkiewicz et al. [44] showed that a combination of SAG1, GRA1, and GRA7 recombinant proteins in an avidity test for IgG antibodies allows for better differentiation between acute and chronic infection in comparison to the lysed, whole-cell antigen. Moreover, paramagnetic microparticles coated with a mixture of SAG1 and GRA8 recombinant antigens have been used in the ARCHITECT Toxo IgG Avidity assay [70]. This avidity test detected 100% of samples drawn less than 4 months after infection as low avidity, thus showing better sensitivity on acute phase specimens than the Vidas Toxo IgG assay (bioMérieux). These results indicate that the ARCHITECT Toxo IgG Avidity assay can not only reliably detect acute phase samples much earlier than the Vidas system, but can also be performed longer for past infection samples with low-level IgG antibodies. Another study reported that the SAG1 recombinant protein mixed with the recombinant GRA7 showed potential for assessing avidity of IgG antibodies [68]. These preliminary results showed better discrimination between acute and chronic infection using the SAG1 + GRA7 combination than those using whole-cell antigens. The recombinant GRA7 cloned from nucleotides 39-711 was also used in an in-house IgG avidity Western blot to discriminate between high and low IgG antibody avidity in sera samples of *T. gondii*-infected patients [65]. Furthermore, Elyasi et al. [64] suggested better performance of avidity ELISA based on GRA6 recombinant antigen than a commercial Euroimmun avidity ELISA for exclusion of a recent infection occurring less than 4 months previously. In 2016, Teh et al. [66] found two novel proteins with diagnostic potential: AG12b encoded *T. gondii* apical complex lysine methyltransferase (AKMT) protein and AG18 encoded *T. gondii* forkhead-associated (FHA) domain-containing protein. These are completely new proteins not belonging to antigen families, such as SAG, GRA, MIC, and ROP groups which were tested in the diagnosis of toxoplasmosis. IgG avidity Western blot and IgG avidity ELISA based on the above-mentioned recombinant proteins were able to differentiate between low avidity and high avidity sera. Thus, rAG12b and rAG18 seem to be useful recombinant proteins for the IgG avidity assays.

## 5. Summary and Conclusions

Because of the great importance of *T. gondii* infection, especially for pregnant women and newborns, the correct diagnosis of the disease is essential. Evaluation of the time of *T. gondii* infection and thus distinction between the early and chronic stages of the disease is crucial. Several serological testing methods, as well as many kinds of commercial tests and fully automatic or semi-automatic systems, are available for detection of specific anti-*T. gondii* antibodies. Many of these serological tests have produced satisfactory results; however, the evaluation of IgG, IgA, and IgM in order to properly diagnose the infection may confront some problems. Since the 1980s, tests measuring the antigen-antibody avidity of IgG against *T. gondii* have been needed in various clinical settings, especially in situations where timing and differentiation of primary and secondary infections are crucial. In recent years, major efforts have been made to improve the ability to distinguish acute from chronic phases of toxoplasmosis defined on the basis of IgG avidity assays. Currently, commercially available immunoassays are mainly based on whole-cell lysate antigens and/or excreted–secreted native antigens. However, many authors have shown that the use of recombinant antigenic preparations in such assays yields improved reproducibility and easier standardization. A growing number of recently conducted studies have demonstrated the diagnostic potential of different forms of recombinant proteins in IgG avidity assays. These antigenic preparations usually produced more mature avidity indices in chronically infected patients and therefore can better distinguish acute and chronic infections. As the avidity test is dependent on the presence of specific IgG, it is very important to use a properly selected recombinant antigen that elicits humoral response at the acute stage of infection. For this reason, many authors focused their attention on the use of proteins that had previously been identified as molecular markers of the early phase of toxoplasmosis in IgG avidity tests. The most frequently studied antigens in avidity tests were SAG1 because it is one of the most immunogenic proteins of *T. gondii* [59], as well as GRA7 and ROP1 because they give a very strong antibody response in the acute phase of infection [74,75]. The other tested antigens mainly belonged to the surface proteins (SAG2), danse granule (GRA3, GRA6, GRA8), and microneme (MIC3, AMA1) families. Although after several years of studying recombinant antigens most of these proteins showed potential for assessing the avidity of specific IgG, no protein has been practically used as a selective marker for discrimination between the recent and late phases of infection. Noteworthy is the fact that the development of specific and reliable approaches for *T. gondii* infection serodiagnosis, which could ideally differentiate between acute and chronic phases of infection, remains very complicated. Unfortunately, the main limitation of some research is the lack of sera from patients with acute phase of toxoplasmosis with a precisely known moment of infection. Such samples of the sera would allow a precise understanding of the maturation of the IgG avidity against various recombinant proteins. Furthermore, several authors have shown that IgG affinity maturation represents a very complex mechanism that may be further modified by hormones developed during pregnancy [76] as well as by treatment with antibiotics [77,78]. Therefore, this should also be considered when the avidity of specific IgG anti-*T. gondii* is determined.

Certainly, the use of recombinant proteins in IgG avidity assays seems to be a very promising resolution for a more precise diagnosis of the disease phase. Many of the tested proteins were found to be potential markers for distinguishing specific antibodies from sera of individuals with the early and chronic phases of *T. gondii* infection. However, further work is needed before an immunoassay with recombinant proteins will be available for clinical purposes.

## Figures and Tables

**Table 1:** Recombinant *T. gondii* antigens tested in the IgG avidity tests.

Antigen	Type of Test	Denaturing Agent	Results	Literature, Year
GRA3, GRA7, MIC3 and SAG1	ELISA	6 M urea	MIC3 antigen—a marker of the early phase of toxoplasmosis. Low avidity index for people infected in the last 2 months.	[60], 2003
ROP1, GRA8, MAG1, SAG1, GRA7	nitrocellulose strips	6 M urea	The research became the basis for the development of the recomLine *Toxoplasma* IgG Avidity diagnostic kit, in which 3 antigens (MAG1, GRA7, SAG1) were used.	[61], 2005
GRA1, GRA7, SAG1	ELISA	6 M urea	The mixture of 3 antigens allows to obtain results similar to those in which Toxoplasma lysate antigen (TLA) was used.	[44], 2007
P30 (SAG1), P35 (GRA8)	ARCHITECT Analyzer and chemiluminescent detection	not specified	IgG avidity test based on SAG1 antigen detected 100% of acute phase specimens (4 months after infection) as low avidity.	[70], 2008
SAG2A	ELISA	6 M urea	SAG2A antigen—suitable for use with the ELISA-IgG1 avidity assay.	[62], 2008
ROP1	ELISA	6 M urea	ROP1 antigen—a potential marker of the early phase of toxoplasmosis.	[63], 2010
GRA6	ELISA	8 M urea	IgG avidity ELISA based on GRA6 antigen was characterized as better performance than commercially available Euroimmun avidity for exclusion of early infection occurring less than 4 months previously.	[64], 2010
rGRA7 cloned from nucleotides 39-711	Western blot	8 M urea	GRA7 recombinant antigen cloned from the gene segment comprising nucleotides 39-711 showed the potential of discrimination between acute and chronic toxoplasmosis	[65], 2013
rROP1, rSAG2 and rGRA6	ELISA	6 M urea	A different trend in avidity maturation of IgG antibodies against two mixture of recombinant antigens (rROP1 + rSAG2 and rROP1 + rGRA6) compression with native antigens was observed. IgG avidity test using these mixtures of antigens may be useful for the diagnosis of difficult-to-identify phases of toxoplasmosis.	[67], 2014
rAG12b and rAG18	Western blot ELISA	8 M urea	rAG12b and rAG18 antigens were able to differentiate low avidity and high avidity serum samples	[66], 2016
Trivalent and tetravalent chimeric proteins: SAG2-GRA1-ROP1, SAG2-GRA1-ROP1-AMA1N, AMA1N-SAG2-GRA1-ROP1, AMA1C-SAG2-GRA1-ROP1, AMA1-SAG2-GRA1-ROP1 (AMA—apical membrane antigen)	ELISA	6 M urea	AMA1-SAG2-GRA1-ROP1 chimeric protein – potential marker for distinguish specific antibodies from sera of individuals with the early and chronic phases *T. gondii* infections	[69], 2019
SAG1 and GRA7	ELISA	8 M urea	SAG1 + GRA7 mixture showed the greatest potential for assessing avidity of antibodies	[68], 2021

## Data Availability

No new data were created or analyzed in this study. Data sharing is not applicable to this article.

## References

[1] Dubey J.P. (2008). The History of *Toxoplasma gondii*—The First 100 Years. J. Eukaryot. Microbiol..

[2] Martínez-Ocampo F. (2018). Genomics of Apicomplexa. Farm Animals Diseases, Recent Omic Trends and New Strategies of Treatment.

[3] Chakraborty S., Roy S., Mistry H.U., Murthy S., George N., Bhandari V., Sharma P. (2015). Potential Sabotage of Host Cell Physiology by Apicomplexan Parasites for Their Survival Benefits. Front. Immunol..

[4] Gubbels M.J., Duraisingh M.T. (2012). Evolution of apicomplexan secretory organelles. Int. J. Parasitol..

[5] Dubey J.P., Weiss L.M., Kim K. (2007). The History and Life Cycle of *Toxoplasma gondii*. Toxoplasma gondii the Model Apicomplexan: Perspectives and Methods.

[6] Dubey J.P., Lindsay D.S., Speer C.A. (1998). Structure of *Toxoplasma gondii* tachyzoites, bradyzoites and sporozoites, and biology and development of tissue cysts. Clin. Microbiol. Rev..

[7] Hunter C.A., Sibley L.D. (2012). Modulation of innate immunity by *Toxoplasma gondii* virulence effectors. Nat. Rev. Microbiol..

[8] Jones J.L., Dubey J.P. (2012). Foodborne Toxoplasmosis. Clin. Infect. Dis..

[9] Tenter A.M., Heckeroth A.R., Weiss L.M. (2000). *Toxoplasma gondii*: From animals to humans. Int. J. Parasitol..

[10] Weiss L.M., Kim K. (2004). The International Congress on Toxoplasmosis. Int. J. Parasitol..

[11] Wang Z.-D., Liu H.-H., Ma Z.-X., Ma H.-Y., Li Z.-Y., Yang Z.-B., Zhu X.-Q., Xu B., Wei F., Liu Q. (2017). *Toxoplasma gondii* Infection in Immunocompromised Patients: A Systematic Review and Meta-Analysis. Front. Microbiol..

[12] Montoya J.G., Remington J.S. (2008). Management of *Toxoplasma gondii* infection during pregnancy. Clin. Infect. Dis..

[13] Ortiz-Alegria L.B., Caballero-Ortega H., Cañedo-Solares I., Rico-Torres C.P., Sahagún-Ruiz A., Medina-Escuti M.E., Correa D. (2010). Congenital toxoplasmosis: Candidate host immune genes relevant for vertical transmission and pathogenesis. Genes Immun..

[14] Zhang K., Lin G., Han Y., Li J. (2016). Serological diagnosis of toxoplasmosis and standardization. Clin. Chim. Acta.

[15] Dard C., Fricker-Hidalgo H., Brenier-Pinchart M.P., Pelloux H. (2016). Relevance of and New Developments in Serology for Toxoplasmosis. Trends Parasitol..

[16] Murat J.B., Hidalgo H.F., Brenier-Pinchart M.P., Pelloux H. (2013). Human toxoplasmosis: Which biological diagnostic tests are best suited to which clinical situations?. Expert Rev. Anti Infect. Ther..

[17] Del Bono V., Canessa A., Bruzzi P., Fiorelli M.A., Terragna A. (1989). Significance of specific immunoglobulin M in the chronological diagnosis of 38 cases of toxoplasmic lymphadenopathy. J. Clin. Microbiol..

[18] Bobic B., Sibalic D., Djurkovic-Djakovic O. (1991). High levels of IgM antibodies specific for *Toxoplasma gondii* in pregnancy 12 years after primary toxoplasma infection. Gynecol. Obstet. Investig..

[19] Liesenfeld O., Montoya J.G., Tathineni N.J., Davis M., Brown B.W., Cobb K.L., Parsonnet J., Remington J.S. (2001). Confirmatory serologic testing for acute toxoplasmosis and rate of induced abortions among women reported to have positive *Toxoplasma* immunoglobulin M antibody titers. Am. J. Obstet. Gynecol..

[20] Liesenfeld O., Press C., Montoya J.G., Gill R., Isaac-Renton J.L., Hedman K., Remington J.S. (1997). False-positive results in immunoglobulin M (IgM) toxoplasma antibody tests and importance of confirmatory testing: The Platelia Toxo IgM test. J. Clin. Microbiol..

[21] Stepick-Biek P., Thulliez P., Araujo F.G., Remington J.S. (1990). IgA antibodies for diagnosis of acute congenital and acquired toxoplasmosis. J. Infect. Dis..

[22] Wong S.Y., Hajdu M.P., Ramirez R., Thulliez P., McLeod R., Remington J.S. (1993). Role of specific immunoglobulin E in diagnosis of acute toxoplasma infection and toxoplasmosis. J. Clin. Microbiol..

[23] Paul M., Goullier-Fleuret A., Pelloux H., Ambroise-Thomas P. (1996). The importance of the detection of anti-P30 IgA antibodies in acquired toxoplasmosis. Acta Parasitol..

[24] Gorgievski-Hrisoho M., Germann D., Matter L. (1996). Diagnostic implications of kinetics of immunoglobulin M and A antibody responses to *Toxoplasma gondii*. J. Clin. Microbiol..

[25] Robert-Gangneux F., Dardé M.L. (2012). Epidemiology of and diagnostic strategies for toxoplasmosis. Clin. Microbiol. Rev..

[26] Reiter-Owona I. (2005). Laboratory diagnosis of toxoplasmosis—Possibilities and limitations. J. Lab. Med..

[27] Petersen E., Borobio M.V., Guy E., Liesenfeld O., Meroni V., Naessens A., Spranzi E., Thulliez P. (2005). European multicenter study of the LIAISON automated diagnostic system for determination of *Toxoplasma gondii*-specific immunoglobulin G (IgG) and IgM and the IgG avidity index. J. Clin. Microbiol..

[28] Jenum P.A., Stray-Pedersen B., Gundersen A.G. (1997). Improved diagnosis of primary Toxoplasma gondii infection in early pregnancy by determination of antitoxoplasma immunoglobulin G avidity. J. Clin. Microbiol..

[29] Cozon G.J., Ferrandiz J., Nebhi H., Wallon M., Peyron F. (1998). Estimation of the avidity of immunoglobulin G for routine diagnosis of chronic *Toxoplasma gondii* infection in pregnant women. Eur. J. Clin. Microbiol. Infect. Dis..

[30] Villard O., Breit L., Cimon B., Franck J., Fricker-Hidalgo H., Godineau N., Houze S., Paris L., Pelloux H., Villena I. (2013). Comparison of four commercially available avidity tests for *Toxoplasma gondii*-specific IgG antibodies. Clin. Vaccine Immunol..

[31] Eisen H.N., Siskind G.W. (1964). Variations in affinities of antibodies during the immune response. Biochemistry.

[32] Werblin T.P., Kim Y.T., Quagliata F., Siskind G.W. (1973). Studies on the control of antibody synthesis. III. Changes in heterogeneity of antibody affinity during the course of the immune response. Immunology.

[33] Hedman K., Lappalainen M., Söderlund M., Hedman L. (1993). Avidity of IgG in serodiagnosis of infectious diseases. Rev. Med. Microbiol..

[34] Hedman K., Lappalainen M., Seppala I., Makela O. (1989). Recent primary toxoplasma infection indicated by a low avidity specific IgG. J. Infect. Dis..

[35] Hedman K., Rousseau S.A. (1989). Measurement of avidity of specific IgG for verification of recent primary rubella. J. Med. Virol..

[36] Hedman K., Seppala I. (1988). Recent rubella virus infection indicated by a low avidity of specific IgG. J. Clin. Immunol..

[37] Grangeot-Keros L., Mayaux M.J., Lebon P., Freymuth F., Eugene G., Stricker R., Dussaix E. (1997). Value of cytomegalovirus (CMV) IgG avidity index for the diagnosis of primary CMV infection in pregnant women. J. Infect. Dis..

[38] Prince H.E., Lapé-Nixon M. (2014). Role of cytomegalovirus (CMV) IgG avidity testing in diagnosing primary CMV infection during pregnancy. Clin. Vaccine Immunol..

[39] Gaudy-Graffin C., Lesage G., Kousignian I., Laperche S., Girault A., Dubois F., Goudeau A., Barin F. (2010). Use of an anti-hepatitis C virus (HCV) IgG avidity assay to identify recent HCV infection. J. Clin. Microbiol..

[40] Junker A.K., Tilley P. (1994). Varicella-zoster virus antibody avidity and IgG-subclass patterns in children with recurrent chickenpox. J. Med. Virol..

[41] Absolom D.R., van Oss C.J. (1986). The nature of the antigen-antibody bond and the factors affecting its association and dissociation. CRC Crit. Rev. Immunol..

[42] Liesenfeld O., Montoya J.G., Kinney S., Press C., Remington J.S. (2001). Effect of testing for IgG avidity in the diagnosis of *Toxoplasma gondii* infection in pregnant women: Experience in a US reference laboratory. J. Infect. Dis..

[43] Paul M. (1999). Immunoglobulin G, avidity in diagnosis of toxolasmic lymphadenopathy and ocular toxoplasmosis. Clin. Diagn. Lab. Immunol..

[44] Pietkiewicz H., Hiszczyńska-Sawicka E., Kur J., Petersen E., Nielsen H.V., Paul M., Stankiewicz M., Myjak P. (2007). Usefulness of *Toxoplasma gondii* recombinant antigens (GRA1, GRA7 and SAG1) in an immunoglobulin G avidity test for the serodiagnosis of toxoplasmosis. Parasitol. Res..

[45] Mechain B., Garin Y.J., Robert-Gangneux F., Dupouy-Camet J., Derouin F. (2000). Lack of utility of specific immunoglobulin G antibody avidity for serodiagnosis of reactivated toxoplasmosis in immunocompromised patients. Clin. Diagn. Lab. Immunol..

[46] Lappalainen M., Hedman K. (2004). Serodiagnosis of toxoplasmosis. The impact of measurement of IgG avidity. Ann. Ist. Super. Sanita..

[47] Holec-Gasior L. (2013). *Toxoplasma gondii* recombinant antigens as tools for serodiagnosis of human toxoplasmosis: Current status of studies. Clin. Vaccine Immunol..

[48] Kotresha D., Noordin R. (2010). Recombinant proteins in the diagnosis of toxoplasmosis. APMIS.

[49] Ferra B., Holec-Gąsior L., Grąźlewska W. (2020). *Toxoplasma gondii* Recombinant Antigens in the Serodiagnosis of Toxoplasmosis in Domestic and Farm Animals. Animals.

[50] Dubremetz J.F., Achbarou A., Bermudes D., Joiner K.A. (1993). Kinetics and pattern of organelle exocytosis during *Toxoplasma gondii*/host-cell interaction. Parasitol. Res..

[51] Carruthers V.B., Sibley L.D. (1997). Sequential protein secretion from three distinct organelles of *Toxoplasma gondii* accompanies invasion of human fibroblasts. Eur. J. Cell Biol..

[52] Carruthers V.B., Giddings O.K., Sibley L.D. (1999). Secretion of micronemal proteins is associated with *Toxoplasma* invasion of host cells. Cell. Microbiol..

[53] Joiner K.A., Roos D.S. (2002). Secretory traffic in the eukaryotic parasite *Toxoplasma gondii*: Less is more. J. Cell Biol..

[54] Ngo H.M., Hoppe H.C., Joiner K.A. (2000). Differential sorting and post-secretory targeting of proteins in parasitic invasion. Trends Cell Biol..

[55] Sam-Yellowe T.Y. (1996). Rhoptry organelles of the Apicomplexa: Their role in host cell invasion and intracellular survival. Parasitol. Today.

[56] Mercier C., Dubremetz J.F., Rauscher B., Lecordier L., Sibley L.D., Cesbron-Delauw M.F. (2002). Biogenesis of nanotubular network in *Toxoplasma* parasitophorous vacuole induced by parasite proteins. Mol. Biol. Cell.

[57] Mercier C., Adjogble K.D., Däubener W., Delauw M.F. (2005). Dense granules: Are they key organelles to help understand the parasitophorous vacuole of all apicomplexa parasites?. Int. J. Parasitol..

[58] Hughes H.P., Van K.F. (1982). Characterization of a secretory antigen from *Toxoplasma gondii* and its role in circulating antigen production. Int. J. Parasitol..

[59] Lekutis C., Ferguson D.J.P., Grigg M.E., Camps M., Boothroyd J.C. (2001). Surface antigens of *Toxoplasma gondii*: Variations of a theme. Int. J. Parasitol..

[60] Beghetto E., Buffolano W., Spadoni A., Del Pezzo M., Di Cristina M., Minenkova O., Petersen E., Felici F., Gargano N. (2003). Use of an immunoglobulin G avidity assay based on recombinant antigens for diagnosis of primary *Toxoplasma gondii* infection during pregnancy. J. Clin. Microbiol..

[61] Pfrepper K.I., Enders G., Gohl M., Krczal D., Hlobil H., Wassenberg D., Soutschek E. (2005). Seroreactivity to and avidity for recombinant antigens in toxoplasmosis. Clin. Diagn. Lab. Immunol..

[62] Béla S.R., Oliveira Silva D.A., Cunha-Júnior J.P., Pirovani C.P., Chaves-Borges F.A., Reis de Carvalho F., Carrijo de Oliveira T., Mineo J.R. (2008). Use of SAG2A recombinant *Toxoplasma gondii* surface antigen as a diagnostic marker for human acute toxoplasmosis: Analysis of titers and avidity of IgG and IgG1 antibodies. Diagn. Microbiol. Infect. Dis..

[63] Holec-Gąsior L., Drapała D., Lautenbach D., Kur J. (2010). *Toxoplasma gondii*: Usefulness of ROP1 recombinant antigen in an immunoglobulin G avidity assay for diagnosis of acute toxoplasmosis in humans. Pol. J. Microbiol..

[64] Elyasi H., Babaie J., Fricker-Hidalgo H., Brenier-Pinchart M.P., Zare M., Sadeghiani G., Assmar M., Pelloux H., Golkar M. (2010). Use of dense granule antigen GRA6 in an immunoglobulin g avidity test to exclude acute *Toxoplasma gondii* infection during pregnancy. Clin. Vaccine Immunol..

[65] Deshpande P.S., Kotresha D., Noordin R., Yunus M.H., Saadatnia G., Golkar M., Osman S., Karim I.Z.A., Ghaffarifar F. (2013). IgG avidity Western blot using *Toxoplasma gondii* rGRA-7 cloned from nucleotides 39-711 for serodiagnosis of acute toxoplasmosis. Rev. Inst. Med. Trop. Sao Paulo.

[66] Teh A.Y., Amerizadeh A., Osman S., Yunus M.H., Noordin R. (2016). Identification, production and assessment of two *Toxoplasma gondii* recombinant proteins for use in a *Toxoplasma* IgG avidity assay. Pathog. Glob. Health.

[67] Drapała D., Holec-Gąsior L., Kur J., Ferra B., Hiszczyńska-Sawicka E., Lautenbach D. (2014). A new human IgG avidity test, using mixtures of recombinant antigens (rROP1, rSAG2, rGRA6), for the diagnosis of difficult-to-identify phases of toxoplasmosis. Diagn. Microbiol. Infect. Dis..

[68] Teimouri A., Afshar M.J.A., Mohtasebi S., Azami S.J., Rasoul Alimi R., Keshavarz H. (2021). Assessment of an In-House Enzyme-Linked Immunosorbent Assay and IgG Avidity Test Based on SAG1 and GRA7 Proteins for Discriminating between Acute and Chronic Toxoplasmosis in Humans. J. Clin. Microbiol..

[69] Ferra B.F., Holec-Gąsior L., Gatkowska J., Dziadek B., Dzitko K., Grąźlewska W., Lautenbach D. (2019). The first study on the usefulness of recombinant tetravalent chimeric proteins containing fragments of SAG2, GRA1, ROP1 and AMA1 antigens in the detection of specific anti-*Toxoplasma gondii* antibodies in mouse and human sera. PLoS ONE.

[70] Sickinger E., Gay-Andrieu F., Jonas G., Schultess J., Stieler M., Smith D., Hausmann M., Stricker R., Stricker R., Dhein J. (2008). Performance characteristics of the new ARCHITECT Toxo IgG and Toxo IgG Avidity assays. Diagn. Microbiol. Infect. Dis..

[71] Marcolino P.T., Silva D.A., Leser P.G., Camargo M.E., Mineo J.R. (2000). Molecular markers in acute and chronic phases of human toxoplasmosis: Determination of immunoglobulin G avidity by Western blotting. Clin. Diagn. Lab. Immunol..

[72] Soldati D., Lassen A., Dubremetz J.F., Boothroyd J.C. (1998). Processing of *Toxoplasma* ROP1 protein in nascent rhoptries. Mol. Biochem. Parasitol..

[73] Bradley P.J., Hsieh C.L., Boothroyd J.C. (2002). Unprocessed *Toxoplasma* ROP1 is effectively targeted and secreted into the nascent parasitophorous vacuole. Mol. Biochem. Parasitol..

[74] Pietkiewicz H., Hiszczyńska-Sawicka E., Kur J., Petersen E., Nielsen H.V., Stankiewicz M., Andrzejewska I., Myjak P. (2004). Usefulness of *Toxoplasma gondii*-specific recombinant antigens in serodiagnosis of human toxoplasmosis. J. Clin. Microbiol..

[75] Holec-Gąsior L., Kur J., Hiszczyńska-Sawicka E. (2009). GRA2 and ROP1 recombinant antigens as potential markers for detection of *Toxoplasma gondii*-specific immunoglobulin G in humans with acute toxoplasmosis. Clin. Vaccine Immunol..

[76] Malan Borel I., Gentile T., Angelucci J., Pividori J., Guala M.D.C., Binaghi R.A., Margni R.A. (1991). IgG asymmetric molecules with antipaternal activity isolated from sera and placenta of pregnant human. J. Reprod. Immunol..

[77] Pelloux H., Brun E., Vernet G., Marcillat S., Jolivet M., Guergour D., Fricker-Hidalgo H., Goullier-Fleuret A., Ambroise-Thomas P. (1998). Determination of anti-*Toxoplasma gondii* immunoglobulin G avidity: Adaptation to the Vidas system (bioMérieux). Diagn. Microbiol. Infect. Dis..

[78] Barberi A., Gistri A., Cappelletti F., Giordano I. (2001). Diagnostic value of IgG avidity in *Toxoplasma* infection: Comparison of 3 commercial kits. J. Infect. Dis..

